# Supplementary material for: Fungistatic Activity Mediated by Volatile Organic Compounds Is Isolate-Dependent in Trichoderma sp. “atroviride B”
Source: J Fungi (Basel). 2023 Feb 10;9(2):238. doi: 10.3390/jof9020238 (PMC9965825; doi:10.3390/jof9020238)
Supplement: Supplementary file 1 [file jof-09-00238-s001.zip › jof-2202419-supplementary.pdf]

Journal of Fungi
MDPI

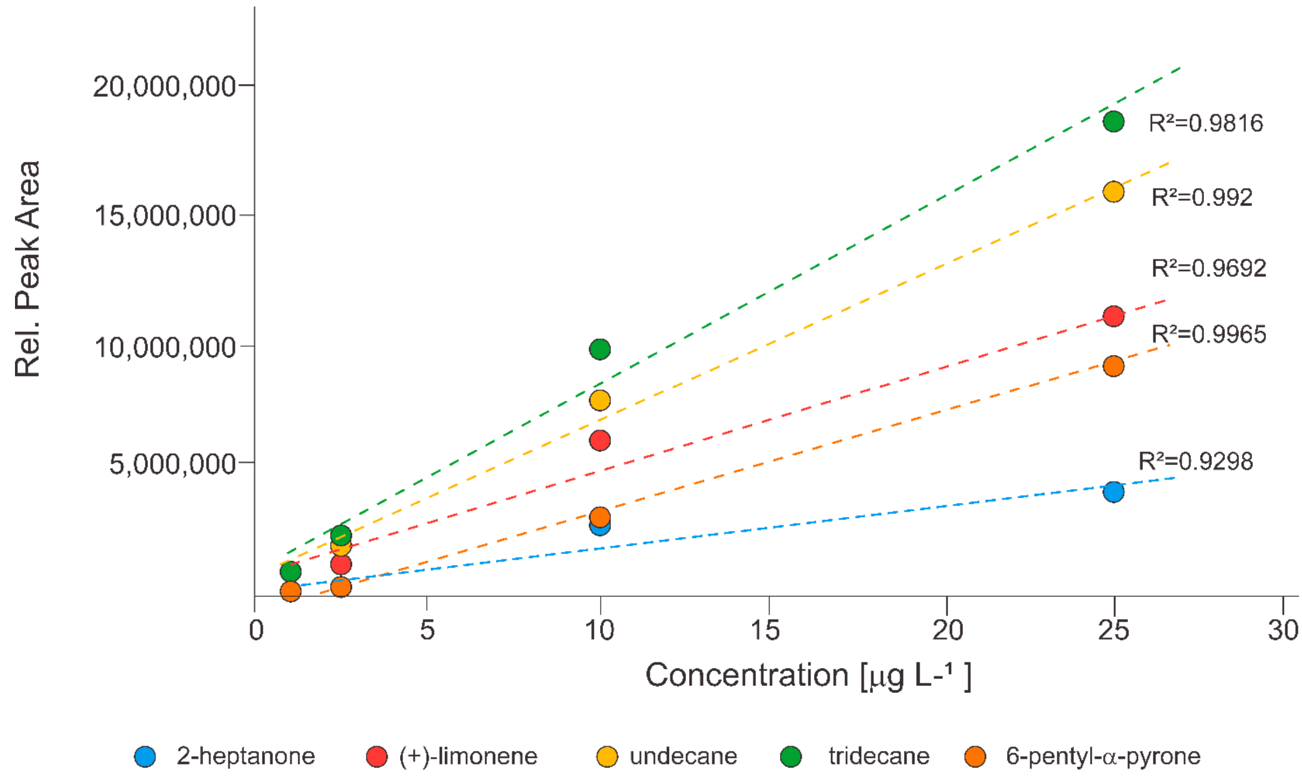

Figure S1. Calibration curves of 5 VOCs detected in the headspace of *Trichoderma* sp. "*atroviride B*" isolates. Mixture of the 5 VOCs were added to 20 mL SPME glass vials in four concentrations (1, 2.5, 10, 25 µg L^{-1}) and analysed with four technical replicates by GC-MS.
